# Characteristics of Men Who Have Sex With Men in Southern Africa Who Seek Sex Online: A Cross-Sectional Study

**DOI:** 10.2196/jmir.4230

**Published:** 2015-05-25

**Authors:** Shauna Stahlman, Ashley Grosso, Sosthenes Ketende, Tampose Mothopeng, Noah Taruberekera, John Nkonyana, Xolile Mabuza, Bhekie Sithole, Zandile Mnisi, Stefan Baral

**Affiliations:** ^1^Center for Public Health and Human RightsDepartment of EpidemiologyJohns Hopkins UniversityBaltimore, MDUnited States; ^2^Matrix Support GroupMaseruLesotho; ^3^Population Services InternationalJohannesburgSouth Africa; ^4^Ministry of HealthMaseruLesotho; ^5^Rock of HopeMbabaneSwaziland; ^6^Ministry of HealthMbabaneSwaziland

**Keywords:** Internet, HIV, male homosexuality, southern Africa, social stigma, sexual behavior

## Abstract

**Background:**

Use of the Internet for finding sexual partners is increasing, particularly among men who have sex with men (MSM). In particular, MSM who seek sex online are an important group to target for human immunodeficiency virus (HIV)/sexually transmitted infection (STI) interventions because they tend to have elevated levels of sexual risk behavior and because the Internet itself may serve as a promising intervention delivery mechanism. However, few studies have examined the correlates of online sexual partner seeking among MSM in sub-Saharan Africa.

**Objective:**

These analyses aim to describe the prevalence of using the Internet to find new male sexual partners among MSM in two southern African countries. In addition, these analyses examine the sociodemographic characteristics, experiences of discrimination and stigma, mental health and substance use characteristics, and HIV-related knowledge, attitudes, and behaviors among MSM associated with meeting sex partners online.

**Methods:**

MSM were enrolled into a cross-sectional study across two sites in Lesotho (N=530), and one in Swaziland (N=322) using respondent-driven sampling. Participants completed a survey and HIV testing. Data were analyzed using bivariate and multivariable logistic regression models to determine which factors were associated with using the Internet to meet sex partners among MSM.

**Results:**

The prevalence of online sex-seeking was high, with 39.4% (209/530) of MSM in Lesotho and 43.8% (141/322) of MSM in Swaziland reporting meeting a new male sexual partner online. In the multivariable analysis, younger age (adjusted odds ratio [aOR] 0.37, 95% confidence interval [CI] 0.27-0.50 per 5 years in Lesotho; aOR 0.68, 95% CI 0.49-0.93 in Swaziland), having more than a high school education (aOR 18.2, 95% CI 7.09-46.62 in Lesotho; aOR 4.23, 95% CI 2.07-8.63 in Swaziland), feeling scared to walk around in public places (aOR 1.89, 95% CI 1.00-3.56 in Lesotho; aOR 2.06, 95% CI 1.23-3.46 in Swaziland), and higher numbers of male anal sex partners within the past 12 months (aOR 1.27, 95% CI 1.01-1.59 per 5 partners in Lesotho; aOR 2.98, 95% CI 1.51-5.89 in Swaziland) were significantly associated with meeting sex partners online in both countries. Additional country-specific associations included increasing knowledge about HIV transmission, feeling afraid to seek health care services, thinking that family members gossiped, and having a prevalent HIV infection among MSM in Lesotho.

**Conclusions:**

Overall, a high proportion of MSM in Lesotho and Swaziland reported meeting male sex partners online, as in other parts of the world. The information in this study can be used to tailor interventions or to suggest modes of delivery of HIV prevention messaging to these MSM, who represent a young and highly stigmatized group. These data suggest that further research assessing the feasibility and acceptability of online interventions will be increasingly critical to addressing the HIV epidemic among MSM across sub-Saharan Africa.

##  Introduction

Globally, the Internet is becoming an increasingly popular platform for meeting new sexual partners, particularly among men who have sex with men (MSM) [1-6]. Recent studies conducted among MSM in Europe and North America indicate that 34-50% report having met a sexual partner online [1,2,4,5]. In particular, MSM who use the Internet to find sexual partners are an important group to target for human immunodeficiency virus (HIV)/sexually transmitted infection (STI) interventions because they tend to have elevated levels of sexual risk behavior and because the Internet itself may serve as a promising intervention delivery mechanism. For example, risk behaviors associated with using the Internet to find sexual partners include increased levels of methamphetamine use [1], higher numbers of sexual partners [1,4,7], and a higher frequency of unprotected anal intercourse [1,4,5,8-10].

In light of these observations, one study measured the acceptability of using Internet-based HIV testing among MSM in Canada and identified perceived benefits to this delivery method including privacy and convenience [11]. Another study conducted in the United States identified high levels of interest for multiple sexual health education topics delivered via the Internet [12]. Further, studies have pointed to the Internet’s ability to enhance the discussion of HIV status and negotiate safer sex practices between partners before meeting [13-15], potentially because the anonymity of the Internet appears to facilitate more direct and less stigmatizing discussions of these complex issues. Finally, additional research suggests that use of the Internet for seeking HIV/STI information is common among MSM using the Internet to find sexual partners [16], further suggesting the potential effectiveness for the Internet to serve as a mechanism to deliver HIV/STI prevention.

However, the majority of research to date assessing the prevalence of, factors associated with, and potential interventions directed towards MSM using the Internet to find sex partners has occurred primarily in higher income settings such as the United States and Europe. Less is known about the Internet sex-seeking behaviors of MSM in sub-Saharan Africa, despite the high and consistent risk for HIV among MSM in this region [17]. In addition, stigma and discrimination may also play a particularly important role in Internet sex-seeking behavior due to high levels of homoprejudice [18-21], which may dissuade MSM from finding sex partners in other venues such as bars or clubs. Of the limited research conducted in sub-Saharan Africa, one study found that using the Internet to find sex partners was associated with testing positive for HIV among MSM in Malawi [21]. Further, having a higher level of education and increased numbers of recent sexual partners were associated with having met sex partners on the Internet among MSM in Cameroon [22]. However, there is a need for additional research to understand the feasibility of Internet-based intervention efforts in sub-Saharan Africa, especially given evidence to suggest that Internet use is growing in the region [23], and particularly among adolescents [24,25].

Because there is evidence suggesting that using the Internet to find sexual partners is associated with risk behaviors among MSM and that the Internet could serve as a promising HIV intervention delivery mechanism, and due to the lack of studies assessing Internet use among MSM in southern Africa, these analyses aim to describe the prevalence of using the Internet to find new male sexual partners online among MSM in two southern African countries. Furthermore, these analyses examine the demographic characteristics, experiences of discrimination and stigma, mental health and substance use characteristics, and HIV-related characteristics among MSM associated with meeting sex partners via the Internet. Taken together, these findings may be used to inform the development of tailored online HIV prevention interventions in southern Africa.

##  Methods

### Study Population and Sampling Methods

Data were collected from February-September 2014 in Maseru and Maputsoe in Lesotho and July-December 2011 in Manzini, Swaziland. Participants were recruited in-person using respondent-driven sampling (RDS) [26,27]. Sampling methods are described in detail elsewhere [28]. In brief, initial recruits or “seeds” recruited peers who then recruited additional peers and so forth, until the desired sample size was reached. Each recruiter could recruit up to three peers. In Maseru, 9 seeds recruited participants through up to 13 waves of accrual, and 7 seeds in Maputsoe recruited participants through up to 17 waves. In Swaziland, 5 seeds recruited participants through up to 14 waves of accrual. In all study sites, equilibrium was reached for several sociodemographic characteristics including age, sexual orientation, and education. Equilibrium was defined using two criteria: (1) close approximation (± 0.03) of the sample proportion and corresponding equilibrium proportion [26], and (2) comparison of the required number of recruitment waves estimated using the respondent driven sampling analysis tool (RDSAT) with the actual number of recruitment waves in the RDS sample [29]. Specifically, the number of recruitment waves estimated to reach equilibrium in RDSAT had to be smaller than the number of waves in the RDS sample for equilibrium to be considered.

MSM were eligible to participate if they were aged 18 years or older, assigned male sex at birth, capable of providing informed consent, and reported having receptive or insertive anal intercourse with another man in the past 12 months. No exclusions were made on the basis of ethnicity, gender identity, or history of HIV testing and diagnosis.

In Swaziland, 4 participants did not answer the question pertaining to meeting partners online and were excluded, resulting in a final sample of 322. All participants in Lesotho answered the question pertaining to meeting partners online (n=530). Participants were reimbursed the equivalent of up to approximately US $10 for their time and travel to the study site. In addition, participants were compensated the equivalent of up to approximately US $2 for each eligible participant they recruited into the study. All participants provided informed oral consent prior to data collection. Data collection was approved by the Institutional Review Board of the Johns Hopkins Bloomberg School of Public Health, the Lesotho National Health Research Ethics Committee, and the Ministry of Health Scientific Ethics Committee in Swaziland.

### Data Collection and Key Measures

During the study visit, trained interviewers administered a structured questionnaire including topics on demographics, social stigma and negative experiences with health care workers and other members of society, social cohesion, mental health and illicit substance use, sexual history, and HIV-related knowledge, attitudes, and behaviors. The primary outcome variable of interest was whether the participant reported meeting new male sexual partners on the Internet. In Lesotho, this was measured by asking, “Where (in what type of place) do you meet new male sexual partners?” Participants were read a list of possible places, and those who responded yes to “online” were indicated as having met male sex partners online. Participants could choose more than one place. In Swaziland, participants were asked, “In the past 12 months, how often have you used the Internet to look for male sexual partners?” and response options were “did not go”, “once a month of less”, “about once a week”, “several times a week”, “about once a day”, and “several times a day”. Those who reported having ever met a partner on the Internet were indicated as having met male sex partners online.

Stigma was measured by a series of “yes” or “no” questions that assessed whether the participant ever felt excluded from family gatherings, that family members gossiped or made discriminatory remarks, rejected by friends, or scared to walk around in public places because of their gender identity or sexual orientation, and if they knew of safe places in their community to socialize with other MSM or had ever been tortured because of their gender identity or sexual orientation. Torture could include both physical abuse such as being beaten or forced to have sex, as well as verbal abuse. In Lesotho, participants were additionally asked if they had ever overheard someone saying discriminatory things about MSM, if they did not feel protected by police, or were ever verbally harassed or blackmailed because of their gender identity or sexual orientation [19-21,30]. Health care stigma was measured by asking participants if they ever avoided (in Lesotho only) or felt afraid to go to health care services because they were worried that someone may learn that they have sex with men, or if they ever felt that they were not treated well in a health center because someone knew they had sex with men.

In Lesotho, depression was assessed using the Patient Health Questionnaire [31], and a positive screen was defined as receiving a score of 10 or greater. In Swaziland, a positive depressive symptoms screen was indicated by answering “yes” to the question, “Have you felt sad or had a depressed mood for more than 2 weeks at a time in the last 3 years?” and suicidal ideation was assessed by asking the participant if he had ever felt like he wanted to end his life. In both countries, drug use was assessed by asking the participant if he had injected any illicit drugs or used any non-injection drugs that were not prescribed to him for health reasons in the past 12 months.

HIV knowledge was assessed by asking participants what type of sex puts them most at risk for HIV, what type of anal sex (ie, insertive or receptive) puts them most at risk for HIV, and what is the safest lubricant to use during anal sex. Attitude towards HIV was measured by asking if they worried about HIV in the past 12 months. Finally, HIV-related behaviors were measured by asking participants if they had ever tested for HIV (in Lesotho) or tested within the past 12 months (in Swaziland), if they had any unprotected anal intercourse (receptive or insertive) in the past 12 months, if they had transactional sex (eg, sex in exchange for drugs or money) with a male partner in the past 12 months, and how many male anal sex partners they had in the past 12 months.

After completing the questionnaire, trained nurse counselors performed blood draws to test for HIV using the Determine Rapid Test (Alere). If tested positive, the sample was then tested using the Unigold Rapid Test (Trinity Biotech). Confirmatory testing was performed for discrepant or indeterminate HIV rapid tests in accordance with national guidelines [32,33].

### Statistical Analysis

RDS-adjusted prevalence estimates for having met a sex partner online at each study site were generated using RDSAT [29]. In a sensitivity analysis, we performed RDS-adjusted bivariate and multivariable logistic regression analyses across each study site by weighting based on the outcome variable. However, because results were convergent and data from the two Lesotho sites were pooled, we present only the unadjusted results [34,35]. Variables were entered into the final logistic multivariable regression model based on a priori knowledge of existing potential confounders and the results of the bivariate analysis. These analyses were performed using SAS software version 9.4.

##  Results

### Sociodemographic Characteristics of Study Sample

The prevalence of seeking partnerships on the Internet was high, with 39.4% (209/530) of MSM in Lesotho and 43.8% (141/322) of MSM in Swaziland reporting meeting a new male sexual partner online (Table 1). Among those who were asked what website they primarily used for meeting partners, the majority in Swaziland reported using Facebook (data not shown). Participants tended to be young overall, with a median age of 23 in Lesotho and 22 in Swaziland. A larger proportion of participants identified as gay or homosexual in Swaziland (202/320, 63.1%) as compared with Lesotho (215/519, 41.4%). In addition, more participants in Lesotho (111/529, 21.0%) reported ever being married to or cohabiting with a female partner as compared with Swaziland (12/318, 3.8%). The median MSM network size was similar across Lesotho (10) and Swaziland (12).

**Table 1:** Prevalence of sociodemographic characteristics and meeting sex partners online among MSM study participants in Lesotho and Swaziland.

Characteristics		Lesotho (N=530)	Swaziland (N=322)
**Age in years, median (interquartile range)**		23 (20-27)	22 (20-26)
**Gender, n (%)**			
	Male	487 (91.9)	232 (72.7)
	Female/intersex	43 (8.1)	87 (27.3)
**Sexual orientation, n (%)**			
	Gay	215 (41.4)	202 (63.1)
	Bisexual	286 (55.1)	114 (35.6)
	Heterosexual	18 (3.5)	4 (1.3)
**Education completed, n (%)**			
	Primary school or less	100 (18.9)	110 (34.2)
	Secondary/High school	347 (65.6)	135 (41.9)
	More than high school	82 (15.5)	77 (23.9)
**Marital status, n (%)**			
	Single/never married	418 (79.0)	306 (96.2)
	Ever married or cohabited	111 (21.0)	12 (3.8)
**Network size, median (interquartile range)**		10 (5-20)	12 (6-30)
**Met male sex partner online, n (%)**		209 (39.4)	141 (43.8)
	RDS-adjusted % (95% CI)	35.7 (28-46)^a^	39.2 (31-49)
	RDS-adjusted % (95% CI)	26.1 (17-36)^b^	

^a^Maseru.

^b^Maputsoe.

### Sociodemographic Characteristics Associated With Meeting a Sex Partner Online

Older age was associated with being less likely to use the Internet to find new male sex partners among MSM in Lesotho (odds ratio [OR] 0.57, 95% confidence interval [CI] 0.47-0.70 per 5 years) (Table 2). In addition, participants who identified as female/intersex were more likely to use the Internet for male sex partners in Lesotho (OR 3.53, 95% CI 1.82-6.86). In both countries, reporting secondary/high school (OR 5.88, 95% CI 3.03-11.39 in Lesotho; OR 2.02, 95% CI 1.19-3.41 in Swaziland) and more than a high school level of education (OR 13.31, 95% CI 6.17-28.73 in Lesotho; OR 2.83, 95% CI 1.54-5.18 in Swaziland) were positively associated with using the Internet to find sex partners. Finally, reporting a larger network of MSM was also associated with using the Internet to find sex partners in Lesotho (OR 1.25, 95% CI 1.12-1.40 per 10 MSM).

**Table 2:** Bivariate associations between sociodemographic characteristics and meeting sex partners online, among MSM in Lesotho and Swaziland.

		Lesotho		Swaziland	
		OR	95% CI	OR	95% CI
**Age, per 5 years**		0.57	0.47-0.70^a^	0.91	0.72-1.15
**Gender**					
	Male	Ref	—	Ref	—
	Female/intersex	3.53	1.82-6.86^a^	1.48	0.90-2.42
**Sexual orientation**					
	Gay	Ref	—	Ref	—
	Bisexual	0.51	0.35-0.73^a^	0.76	0.48-1.21
	Heterosexual^b^	0.20	0.06-0.70^a^	—	—
**Education completed**					
	Primary school or less	Ref	—	Ref	—
	Secondary/High school	5.88	3.03-11.39^a^	2.02	1.19-3.41^a^
	More than high school	13.31	6.17-28.73^a^	2.83	1.54-5.18^a^
**Marital status**					
	Single/never married	Ref	—	Ref	—
	Ever married or cohabited	0.76	0.49-1.18	0.93	0.29-2.99
**Network size, per 10 MSM**		1.25	1.12-1.40^a^	1.03	0.99-1.07

^a^ *P*<.05.

^b^Sample size was not large enough to generate OR in Swaziland.

### Social and Health Care Stigma and Mental Health Factors

In Lesotho, meeting new male sex partners online was significantly associated with feeling that family members gossiped or made discriminatory remarks about the participant’s sexual orientation or gender identity (OR 4.86, 95% CI 2.80-8.43), feeling excluded by family members (OR 2.53, 95% CI 1.29- 4.97), hearing discriminatory remarks about MSM (OR 2.34, 95% CI 1.48-3.72), being verbally harassed (OR 2.48, 95% CI 1.73-3.55), feeling scared to walk around in public (OR 3.20, 95% CI 1.94-5.30), and being tortured (OR 4.17, 95% CI 1.46-11.86) (Table 3). In Swaziland, feeling rejected by friends (OR 2.17, 95% CI 1.38-3.41) and feeling scared to walk around in public (OR 1.79, 95% CI 1.14-2.79) were associated with meeting a new male sex partner online. Finally, meeting sex partners online was associated with being afraid to seek health care services (OR 3.32, 95% CI 1.91-5.76), avoiding services (OR 2.43, 95% CI 1.28-4.62), and being treated poorly by a health care worker (OR 3.48, 95% CI 1.30-9.31) in Lesotho.

There was only one statistically significant association detected between mental health and substance use measures and meeting sex partners online (Table 4). In Lesotho, screening positive for depression was associated with using the Internet to meet sex partners (OR 1.77, 95% CI 1.11-2.83).

**Table 3:** Bivariate associations between stigma and meeting sex partners online, among MSM in Lesotho and Swaziland.

Explanatory variable		Lesotho			Swaziland		
		n^a^	OR	95% CI	n^a^	OR	95% CI
**Social stigma**							
	Family exclusion	38	2.53	1.29-4.97^b^	81	1.11	0.67-1.84
	Family gossiped	71	4.86	2.80-8.43^b^	157	1.39	0.90-2.17
	Friend rejection	100	1.33	0.86-2.06	176	2.17	1.38-3.41^b^
	No safe place to socialize with other MSM	160	1.05	0.72-1.54	200	1.09	0.69-1.73
	Heard discriminatory remarks about MSM	413	2.34	1.48-3.72^b^	—	—	—
	Did not feel protected by police	18	2.50	0.96-6.57	—	—	—
	Felt scared to walk around in public	77	3.20	1.94-5.30^b^	145	1.79	1.14-2.79^b^
	Verbally harassed	214	2.48	1.73-3.55^b^	—	—	—
	Blackmailed	100	0.93	0.59-1.45	—	—	—
	Tortured	18	4.17	1.46-11.86^b^	129	1.20	0.77-1.88
**Health care stigma**							
	Afraid to seek services	63	3.32	1.91-5.76^b^	177	1.07	0.69-1.67
	Avoided services	42	2.43	1.28-4.62^b^	—	—	—
	Treated poorly	19	3.48	1.30-9.31^b^	54	0.86	0.48-1.56

^a^n refers to the number of participants who indicated “yes” for the explanatory variable.

^b^ *P*<.05.

### HIV Knowledge, Attitudes, and Behaviors and Prevalent HIV Infection

Overall, a greater knowledge about HIV transmission was associated with meeting sex partners online (Table 4). In particular, knowing that water-based lubrication is the safest to use during anal sex was significantly associated with finding partners online in both countries (OR 3.01, 95% CI 2.09-4.32 in Lesotho; OR 1.66, 95% CI 1.05-2.61 in Swaziland). In addition, knowing the correct answer to all three questions assessing HIV transmission knowledge was associated with meeting partners online in Lesotho (OR 2.72, 95% CI 1.55-4.79). Among those who had never been diagnosed with HIV, being worried about HIV infection was significantly associated with meeting partners online in Lesotho (OR 1.69, 95% CI 1.04-2.76).

There were no associations detected between meeting sex partners on the Internet and ever receiving an HIV test, reporting any recent unprotected anal intercourse, or engaging in transactional sex in either country. However, across both countries reporting a greater number of male anal sex partners in the past 12 months was associated with meeting sex partners online (OR 1.30, 95% CI 1.07-1.56 per 5 partners in Lesotho; OR 2.24, 95% CI 1.20-4.19 per 5 partners in Swaziland). Finally, testing positive for HIV was significantly associated with having met partners online in Lesotho (OR 1.50, 95% CI 1.04-2.17).

**Table 4:** Bivariate associations between HIV-related variables and meeting sex partners online, among MSM in Lesotho and Swaziland.

Explanatory variable			Lesotho			Swaziland		
			n^a^	OR	95% CI	n^a^	OR	95% CI
**Mental health**								
	Depressed		84	1.77	1.11-2.83^b^	208	1.18	0.74-1.87
	Suicidal ideation, ever		—	—	—	141	1.37	0.88-2.13
**Substance use**								
	Any illicit drug use, past 12 months		89	0.79	0.49-1.28	113	1.52	0.96-2.40
**HIV-related knowledge, attitudes, and behaviors**								
		Anal sex is riskier than vaginal or oral sex	138	2.28	1.54-3.39^b^	80	0.87	0.52-1.44
		Receptive anal sex is riskier than insertive	267	0.78	0.55-1.11	96	1.00	0.62-1.62
		Water-based lubricant is the safest to use during anal sex	219	3.01	2.09-4.32^b^	134	1.66	1.05-2.61^b^
		All of the above	57	2.72	1.55-4.79^b^	23	0.81	0.34, 1.94
	Worried about HIV^c^		84	1.69	1.04-2.76^b^	150	1.14	0.72-1.82
	Tested for HIV^d^		366	1.57	0.97-2.56	161	1.08	0.68-1.70
	Any unprotected AI, past 12 months		267	1.34	0.95-1.91	143	1.31	0.81-2.12
	Transactional sex with male, past 12 months		170	1.11	0.77-1.61	84	0.83	0.50-1.38
	Five additional male AI partners, past 12 months, median (IQR)		3 (1-4)	1.30	1.07-1.56^b^	2 (1-3)	2.24	1.20-4.19^b^
**Laboratory testing**								
	Positive for HIV		173	1.50	1.04-2.17	55	1.07	0.60-1.92

^a^n refers to the number of participants who indicated “yes” for the explanatory variable.

^b^ *P*<.05.

^c^Among those who have never been told that they have HIV.

^d^Refers to ever in Lesotho and past 12 months in Swaziland.

### Factors Independently Associated With Meeting Sex Partners Online

In the multivariable analysis, younger age, increased level of education, feeling scared to walk around in public places, and higher numbers of male anal sex partners were significantly associated with meeting sex partners online in both countries (Table 5). Additional country-specific associations included increasing knowledge about HIV transmission (adjusted odds ratio [aOR] 1.29, 95% CI 1.02-1.64), feeling afraid to seek health care services (aOR 2.31, 95% CI 1.15-4.65), feeling that family members gossiped (aOR 3.42, 95% CI 1.76-6.67), and having a prevalent HIV infection (aOR 2.45, 95% CI 1.41-4.24) in Lesotho.

**Table 5:** Factors independently associated with meeting sex partners online, among MSM in Lesotho and Swaziland (variables entered into single logistic regression model for each country).

Explanatory variable	Lesotho		Swaziland	
	aOR	95% CI	aOR	95% CI
Age, per 5 years	0.37	0.27-0.50^a^	0.68	0.49-0.93^a^
Secondary/High school	6.15	2.74-13.78^a^	2.46	1.38-4.39^a^
More than high school	18.18	7.09-46.62^a^	4.23	2.07-8.63^a^
Female/other gender	2.03	0.87-4.74	1.50	0.85-2.65
Ever married or cohabited	2.15	1.23-3.76^a^	1.77	0.49-6.43
Ever felt scared to walk around in public	1.89	1.00-3.56^a^	2.06	1.23-3.46^a^
Ever felt that family members gossiped	3.42	1.76-6.67^a^	1.29	0.79-2.11
Ever felt afraid to go to health care services	2.31	1.15-4.65^a^	0.98	0.58-1.64
Increasing knowledge of HIV transmission^b^	1.29	1.02-1.64^a^	1.01	0.76-1.35
Five additional male AI partners, past 12 months	1.27	1.01-1.59^a^	2.98	1.51-5.89^a^
Tested positive for HIV	2.45	1.41-4.24^a^	1.39	0.68-2.84

^a^ *P*<.05.

^b^Refers to number of HIV-related knowledge questions answered correctly.

##  Discussion

### Principal Findings

In this study of MSM across two countries in southern Africa, we found that a high proportion reported using the Internet to meet new male sex partners, and the prevalence was similar to that observed among MSM across North America and Europe [1,2,4,5]. Southern African MSM who reported social and health care stigma, were younger, more educated, were knowledgeable about HIV transmission, and reported a greater number of recent anal sex partners were more likely to report using the Internet to meet male sex partners. Within Lesotho, MSM who had larger MSM networks screened positive for depression and tested positive for HIV were also more likely to meet partners online. These findings have implications for the future development of Internet-based HIV interventions.

Recent online HIV intervention efforts directed towards MSM are summarized in a 2007 literature review [36]. These efforts include feasibility studies of Internet- and mobile phone-based programs primarily designed to reduce HIV risk behavior, with overall high levels of feasibility noted thus far. However, most of this work has been conducted in the United States or Europe with limited focus on sub-Saharan Africa. One likely significant difference between populations of MSM in higher income settings as compared to MSM in sub-Saharan Africa is the level of stigma and discrimination. In particular, male homosexual acts are criminalized in Swaziland, and in Lesotho sodomy is still prohibited as a common-law offense [37]. Not only are stigma and discrimination likely to be higher in these countries, but these results suggest that those MSM who use the Internet to meet sex partners report even higher levels of stigma and discrimination than their peers. This suggests that there may be opportunities via Internet-based methods to link these individuals to social support networks, including cyber-support networks, to help increase skills for coping with stigma. For example, one study conducted among racial/ethnic minority MSM in Los Angeles, CA, indicated that social media-based HIV prevention interventions can increase community cohesion and facilitate discussions about HIV-related stigma and discrimination [38,39]. In contrast, because reporting a larger MSM network was also associated with Internet sex-seeking in Lesotho, existing Internet social networks could be used to deliver HIV interventions or to facilitate linkage to HIV testing and care in health care venues that are sensitive to the needs of MSM. The finding that larger sexual networks are associated with using the Internet to meet new sex partners has been identified previously by studies in higher income settings [1,8,40] and indicates that much of the knowledge attained from these studies might be applicable to Internet-using MSM in southern Africa. However, additional feasibility studies are needed for Internet-based HIV interventions among MSM in sub-Saharan Africa, including where and how these men access the Internet.

The finding that younger, more educated and informed individuals were using the Internet to meet sex partners may be reflective of the population subgroups who have better access to the Internet in general. However, the Internet may be particularly relevant to younger MSM in building and establishing peer networks that support sexual health and empowerment, in part because adolescence is an important time for development of self-acceptance and sexual identity [4,41,42]. Because of the positive association between existing HIV transmission knowledge and Internet sex-seeking, Internet-based interventions to increase HIV knowledge may be less effective for MSM using the Internet to find partners. Instead, efforts might be better focused towards the Internet’s ability to facilitate safer sex negotiation and serostatus disclosure [13,14]. For example, MSM could be encouraged to disclose their HIV status on their online profile or to discuss their HIV status and preferences for condom use with potential sex partners before meeting. However, there is some concern for the potential misrepresentation of HIV status among those who have never tested or have tested positive, possibly as a result of HIV stigma or fear of losing sexual partners [43]. Because the Internet allows for the opportunity to interact with greater numbers of sexual partners, safer sex negotiation may be even more important as the opportunity for exposure to HIV and STIs is increased.

In addition, these findings suggest some variability by country. For example, testing positive for HIV was associated with meeting sex partners online among MSM in Lesotho but not in Swaziland. This speaks to the importance of tailoring online interventions for different groups of MSM by region, as MSM in one region may not have the same experiences or needs as MSM in other parts of southern Africa. However, we did find consistency in several of our results across countries, suggesting that MSM who use the Internet to find sex partners are a relatively defined group with specific characteristics and needs.

### Limitations

There are several limitations of these analyses. First, data were cross-sectional and as such we make no effort to infer causality. Instead, our objective was to describe the prevalence of and characteristics associated with meeting a sex partner online among MSM in southern Africa because they represent an important understudied group with many untapped opportunities for intervention. Second, comparisons made across countries must be interpreted with caution as there was some variability in the phrasing of the question regarding Internet use to find sex partners. In particular, the Lesotho questionnaire asked about current online partner seeking and the Swaziland questionnaire asked about online partner seeking within the past 12 months. In addition, data were collected in 2014 in Lesotho and 2011 in Swaziland, which could account for some variation between countries because the Internet was less popular and more expensive in 2011 as compared with 2014 in this region. However, there were similarities in the response distribution and measures of associations between the two countries, which suggests that both questions similarly measured recent Internet use to find male sex partners.

Finally, the use of RDS requires certain underlying assumptions to be met [44]. Because of the difficulty involved with testing these assumptions, it is possible that not all assumptions were not met in this study. For example, participants were not explicitly asked during the interview how they knew their recruiter. However, recruiters were instructed to recruit only MSM who they knew and who also knew them. Further, because there are limited data available regarding the total number of MSM in Lesotho and Swaziland, it is difficult to test the assumption that the recruited sample size is small relative to the overall size of the target population. In addition, there can be bias introduced in RDS by the non-random selection of individuals out of a recruiter’s social network [45]. However, results from our RDS-adjusted sensitivity analysis indicated similar results to the unadjusted estimates presented here. Because equilibrium was reached for several sociodemographic characteristics in this study, this further suggests a minimal overall bias due to non-random recruitment.

### Conclusions

Overall, a high proportion of MSM in southern Africa report meeting male sex partners online, as in other parts of the world. The information in this study can be used to tailor interventions to young, educated, and stigmatized MSM who were most likely to report using the Internet to find sex partners. Internet use for meeting sexual partners is likely to increase in prevalence in coming years among MSM in southern Africa, as it is currently common among younger age groups. These trends reinforce the potential value of engaging MSM in these settings through online interventions. Moreover, interventions should draw from the lessons of online engagement of MSM in other parts of the world including North America, Europe, and Asia given the similarities observed. Ultimately, these analyses suggest that further research assessing the feasibility and acceptability of online interventions will be increasingly critical to addressing the HIV epidemic among MSM in sub-Saharan Africa.

## Abbreviations

AI: anal intercourse
aOR: adjusted odds ratio
HIV: human immunodeficiency virus
IQR: interquartile range
MSM: men who have sex with men
OR: odds ratio
RDS: respondent-driven sampling
RDSAT: respondent-driven sampling analysis tool
STI: sexually transmitted infection

## References

[1] Benotsch Eric G, Kalichman Seth, Cage Maggi (2002). Men who have met sex partners via the Internet: prevalence, predictors, and implications for HIV prevention. Arch Sex Behav.

[2] Bolding Graham, Davis Mark, Hart Graham, Sherr Lorraine, Elford Jonathan (2005). Gay men who look for sex on the Internet: is there more HIV/STI risk with online partners?. AIDS.

[3] Glick Sara Nelson, Morris Martina, Foxman Betsy, Aral Sevgi O, Manhart Lisa E, Holmes King K, Golden Matthew R (2012). A comparison of sexual behavior patterns among men who have sex with men and heterosexual men and women. J Acquir Immune Defic Syndr.

[4] Garofalo Robert, Herrick Amy, Mustanski Brian S, Donenberg Geri Rachel (2007). Tip of the Iceberg: young men who have sex with men, the Internet, and HIV risk. Am J Public Health.

[5] Liau Adrian, Millett Gregorio, Marks Gary (2006). Meta-analytic examination of online sex-seeking and sexual risk behavior among men who have sex with men. Sex Transm Dis.

[6] Chiasson Mary Ann, Hirshfield Sabina, Remien Robert H, Humberstone Mike, Wong Tom, Wolitski Richard J (2007). A comparison of on-line and off-line sexual risk in men who have sex with men: an event-based on-line survey. J Acquir Immune Defic Syndr.

[7] Horvath Keith J, Rosser B R Simon, Remafedi Gary (2008). Sexual risk taking among young internet-using men who have sex with men. Am J Public Health.

[8] Blackwell Christopher W (2008). Men who have sex with men and recruit bareback sex partners on the internet: implications for STI and HIV prevention and client education. Am J Mens Health.

[9] Lewnard Joseph A, Berrang-Ford Lea (2014). Internet-based partner selection and risk for unprotected anal intercourse in sexual encounters among men who have sex with men: a meta-analysis of observational studies. Sex Transm Infect.

[10] Yang Zhongrong, Zhang Sichao, Dong Zhengquan, Jin Meihua, Han Jiankang (2014). Prevalence of unprotected anal intercourse in men who have sex with men recruited online versus offline: a meta-analysis. BMC Public Health.

[11] Gilbert Mark, Hottes Travis Salway, Kerr Thomas, Taylor Darlene, Fairley Christopher K, Lester Richard, Wong Tom, Trussler Terry, Marchand Rick, Shoveller Jean, Ogilvie Gina (2013). Factors associated with intention to use internet-based testing for sexually transmitted infections among men who have sex with men. J Med Internet Res.

[12] Hooper Simon, Rosser B R Simon, Horvath Keith J, Oakes J Michael, Danilenko Gene, Men's INTernet Sex II (MINTS-II) Team (2008). An online needs assessment of a virtual community: what men who use the internet to seek sex with men want in Internet-based HIV prevention. AIDS Behav.

[13] Mustanski Brian, Lyons Tom, Garcia Steve C (2011). Internet use and sexual health of young men who have sex with men: a mixed-methods study. Arch Sex Behav.

[14] Carballo-Diéguez Alex, Miner Michael, Dolezal Curtis, Rosser B R Simon, Jacoby Scott (2006). Sexual negotiation, HIV-status disclosure, and sexual risk behavior among Latino men who use the internet to seek sex with other men. Arch Sex Behav.

[15] Wohlfeiler Dan, Potterat John J (2005). Using gay men's sexual networks to reduce sexually transmitted disease (STD)/human immunodeficiency virus (HIV) transmission. Sex Transm Dis.

[16] Rietmeijer Cornelis A, Bull Sheana S, McFarlane Mary, Patnaik Jennifer Landrigan, Douglas John M (2003). Risks and benefits of the internet for populations at risk for sexually transmitted infections (STIs): results of an STI clinic survey. Sex Transm Dis.

[17] Baral Stefan, Sifakis Frangiscos, Cleghorn Farley, Beyrer Chris (2007). Elevated risk for HIV infection among men who have sex with men in low- and middle-income countries 2000-2006: a systematic review. PLoS Med.

[18] Risher Kathryn, Adams Darrin, Sithole Bhekie, Ketende Sosthenes, Kennedy Caitlin, Mnisi Zandile, Mabusa Xolile, Baral Stefan D (2013). Sexual stigma and discrimination as barriers to seeking appropriate healthcare among men who have sex with men in Swaziland. J Int AIDS Soc.

[19] Wirtz Andrea L, Jumbe Vincent, Trapence Gift, Kamba Dunker, Umar Eric, Ketende Sosthenes, Berry Mark, Strömdahl Susanne, Beyrer Chris, Baral Stefan D (2013). HIV among men who have sex with men in Malawi: elucidating HIV prevalence and correlates of infection to inform HIV prevention. J Int AIDS Soc.

[20] Baral Stefan, Adams Darrin, Lebona Judith, Kaibe Bafokeng, Letsie Puleng, Tshehlo Relebohile, Wirtz Andrea, Beyrer Chris (2011). A cross-sectional assessment of population demographics, HIV risks and human rights contexts among men who have sex with men in Lesotho. J Int AIDS Soc.

[21] Baral Stefan, Trapence Gift, Motimedi Felistus, Umar Eric, Iipinge Scholastika, Dausab Friedel, Beyrer Chris (2009). HIV prevalence, risks for HIV infection, and human rights among men who have sex with men (MSM) in Malawi, Namibia, and Botswana. PLoS One.

[22] Henry Emilie, Yomb Yves, Fugon Lionel, Spire Bruno (2012). The use of the Internet in male sexual encounters by men who have sex with men in Cameroon. Digital Culture & Education.

[23] International Telecommunication Union (2012). ITU Study Group 3.

[24] Ybarra Michele L, Kiwanuka Julius, Emenyonu Nneka, Bangsberg David R (2006). Internet use among Ugandan adolescents: implications for HIV intervention. PLoS Med.

[25] Borzekowski Dina L G, Fobil Julius N, Asante Kofi O (2006). Online access by adolescents in Accra: Ghanaian teens' use of the internet for health information. Dev Psychol.

[26] Heckathorn DD (1997). Respondent-Driven Sampling: A New Approach to the Study of Hidden Populations. Social Problems.

[27] Magnani Robert, Sabin Keith, Saidel Tobi, Heckathorn Douglas (2005). Review of sampling hard-to-reach and hidden populations for HIV surveillance. AIDS.

[28] Baral Stefan D, Ketende Sosthenes, Mnisi Zandile, Mabuza Xolile, Grosso Ashley, Sithole Bhekie, Maziya Sibusiso, Kerrigan Deanna L, Green Jessica L, Kennedy Caitlin E, Adams Darrin (2013). A cross-sectional assessment of the burden of HIV and associated individual- and structural-level characteristics among men who have sex with men in Swaziland. J Int AIDS Soc.

[29] Volz E, Wejnert C, Cameron C, Spiller M, Barash V, Degani I, Heckathorn DD (2012). Ithaca, NY: Cornell University.

[30] Fay Heather, Baral Stefan D, Trapence Gift, Motimedi Felistus, Umar Eric, Iipinge Scholastika, Dausab Friedel, Wirtz Andrea, Beyrer Chris (2011). Stigma, health care access, and HIV knowledge among men who have sex with men in Malawi, Namibia, and Botswana. AIDS Behav.

[31] Kroenke K, Spitzer R L, Williams J B (2001). The PHQ-9: validity of a brief depression severity measure. J Gen Intern Med.

[32] (2007). Ministry of Health and Social Welfare.

[33] (2010). Ministry of Health.

[34] Heckathorn DD (2007). Extensions of respondent-driven sampling: Analyzing continuous variables and controlling for differential recruitment. Sociological Methodology.

[35] Winship C, Radbill L (1994). Sampling Weights and Regression Analysis. Sociological Methods & Research.

[36] Ybarra Michele L, Bull Sheana S (2007). Current trends in Internet- and cell phone-based HIV prevention and intervention programs. Curr HIV/AIDS Rep.

[37] Itaborahy LP, Zhu J (2014). A world survey of laws: Criminalisation, protection and recognition of same-sex love.

[38] Young Sean D, Holloway Ian, Jaganath Devan, Rice Eric, Westmoreland Drew, Coates Thomas (2014). Project HOPE: online social network changes in an HIV prevention randomized controlled trial for African American and Latino men who have sex with men. Am J Public Health.

[39] Young Sean D, Jaganath Devan (2013). Online social networking for HIV education and prevention: a mixed-methods analysis. Sex Transm Dis.

[40] Lehmiller Justin J, Ioerger Michael (2014). Social networking smartphone applications and sexual health outcomes among men who have sex with men. PLoS One.

[41] McFarlane Mary, Bull Sheana S, Rietmeijer Cornelis A (2002). Young adults on the Internet: risk behaviors for sexually transmitted diseases and HIV(1). J Adolesc Health.

[42] Mustanski Brian S, Newcomb Michael E, Du Bois Steve N, Garcia Steve C, Grov Christian (2011). HIV in young men who have sex with men: a review of epidemiology, risk and protective factors, and interventions. J Sex Res.

[43] Horvath Keith J, Oakes J Michael, Rosser B R Simon (2008). Sexual negotiation and HIV serodisclosure among men who have sex with men with their online and offline partners. J Urban Health.

[44] Lansky Amy, Drake Amy, Wejnert Cyprian, Pham Huong, Cribbin Melissa, Heckathorn Douglas D (2012). Assessing the assumptions of respondent-driven sampling in the national HIV Behavioral Surveillance System among injecting drug users. Open AIDS J.

[45] Wejnert C (2009). An empirical test of respondent-driven sampling: Point estimates, variance, degree measures, and out-of-equilibrium data. Sociological Methodology.

